# PEACE V – Salvage Treatment of OligoRecurrent nodal prostate cancer Metastases (STORM): a study protocol for a randomized controlled phase II trial

**DOI:** 10.1186/s12885-020-06911-4

**Published:** 2020-05-12

**Authors:** A. De Bruycker, A. Spiessens, P. Dirix, N. Koutsouvelis, I. Semac, N. Liefhooghe, A. Gomez-Iturriaga, W. Everaerts, F. Otte, A. Papachristofilou, M. Scorsetti, M. Shelan, S. Siva, F. Ameye, M. Guckenberger, R. Heikkilä, P. M. Putora, A. Zapatero, A. Conde-Moreno, F. Couñago, F. Vanhoutte, E. Goetghebeur, D. Reynders, T. Zilli, P. Ost

**Affiliations:** 1 Department of Radiation oncology and experimental cancer research, Ghent University Hospital, Ghent, Belgium; 2 Department of Radiation oncology, Iridium Cancer Network, GZ Antwerp, Antwerp, Belgium; 3 Department of Radiation oncology, Geneva University Hospital, Geneva, Switzerland; 4 Clinical Research Center, Geneva University Hospital and Faculty of Medicine, Geneva, Switzerland; 5 Department of Radiation oncology, AZ Groeninge, Kortrijk, Belgium; 6 Cruces University Hospital (Biocruces Health Research Institute), Barakaldo, Spain; 7 Department of Development and Regeneration, KU Leuven, Leuven, Belgium; 8 Department of Radiation oncology, Jules Bordet Institute and Hôpital Erasme, University Clinics of Brussels, Université Libre de Bruxelles, Brussels, Belgium; 9 Clinic of Radiotherapy & Radiation Oncology, University Hospital Basel, Basel, Switzerland; 10 Humanitas Clinical and Research Hospital, IRCSS, Radiotherapy and Radiosurgery Department, Rozzano, Milan, Italy; 11 Department of Radiation oncology, Inselspital, Bern University Hospital, University of Bern, Bern, Switzerland; 12 Epworth Healthcare, University of Melbourne, Melbourne, Australia; 13 Department of Urology, AZ Maria-Middelares Ghent, Ghent, Belgium; 14 Department of Radiation Oncology, University Hospital Zürich, University of Zurich, Zürich, Switzerland; 15 Department of Oncology, Oslo University Hospital, Oslo, Norway; 16 Department of Radiation Oncology, Kantonsspital St. Gallen, St. Gallen, Switzerland; 17 University Hospital La Princesa, Madrid, Spain; 18 Department of Radiation oncology, Hospital Universitari i Politècnic la Fe, Valencia, Spain; 19 Department of Radiation oncology, University Hospital of Quirón, Madrid, Spain; 20 Department of Applied Mathematics, Computer Science and Statistics, Ghent University, Ghent, Belgium

**Keywords:** Prostate cancer, Oligometastases, Oligorecurrence, Stereotactic body radiotherapy, Whole pelvic radiotherapy, Salvage lymph node dissection, Metastasis-directed therapy, Quality of life, Survival, Androgen deprivation therapy

## Abstract

**Background:**

Pelvic nodal recurrences are being increasingly diagnosed with the introduction of new molecular imaging techniques, like choline and PSMA PET-CT, in the restaging of recurrent prostate cancer (PCa). At this moment, there are no specific treatment recommendations for patients with limited nodal recurrences and different locoregional treatment approaches are currently being used, mostly by means of metastasis-directed therapies (MDT): salvage lymph node dissection (sLND) or stereotactic body radiotherapy (SBRT). Since the majority of patients treated with MDT relapse within 2 years in adjacent lymph node regions, with an estimated median time to progression of 12–18 months, combining MDT with whole pelvic radiotherapy (WPRT) may improve oncological outcomes in these patients. The aim of this prospective multicentre randomized controlled phase II trial is to assess the impact of the addition of WPRT to MDT and short-term androgen deprivation therapy (ADT) on metastasis-free survival (MFS) in the setting of oligorecurrent pelvic nodal recurrence.

**Methods & design:**

Patients diagnosed with PET-detected pelvic nodal oligorecurrence (≤5 nodes) following radical local treatment for PCa, will be randomized in a 1:1 ratio between arm A: MDT and 6 months of ADT, or arm B: WPRT added to MDT and 6 months of ADT. Patients will be stratified by type of PET-tracer (choline, FACBC or PSMA) and by type of MDT (sLND or SBRT). The primary endpoint is MFS and the secondary endpoints include clinical and biochemical progression-free survival (PFS), prostate cancer specific survival, quality of life (QoL), toxicity and time to castration-resistant prostate cancer (CRPC) and to palliative ADT. Estimated study completion: December 31, 2023.

**Discussion:**

This is the first prospective multicentre randomized phase II trial assessing the potential of combined WPRT and MDT as compared to MDT alone on MFS for patients with nodal oligorecurrent PCa.

**Trial registration:**

ClinicalTrials.gov Identifier: NCT03569241, registered June 14, 2018, ;

Identifier on Swiss National Clinical Trials Portal (SNCTP): SNCTP000002947, registered June 14, 2018.

## Background

A proportion of prostate cancer (PCa) patients develop a local, regional (N1) or distant (M1) relapse following curative local treatment. For both local and distant relapse, different treatment recommendations are made in the EAU-EANM-ESTRO-ESUR-SIOG guidelines on Prostate Cancer [1]. The entity of regional nodal recurrence is not specifically mentioned in the guidelines although it is an emerging clinical situation with the introduction of new molecular imaging techniques like choline and more recently PSMA PET-CT in the restaging of recurrent PCa [2]. More specifically, a subgroup of these patients is being diagnosed with a recurrence confined to the regional lymph nodes and limited in number (oligorecurrence) using metabolic imaging [3, 4]. As there are no specific treatment recommendations for limited metastatic disease, different treatment approaches are currently used, mostly focusing on local ablative treatment using radiotherapy or surgery [5–8]. These treatments are coined metastasis-directed therapy (MDT) [5]. MDT, whether or not in combination with temporary androgen deprivation therapy (ADT), has the potential to reduce the subsequent risk of progression or even to cure limited regional nodal recurrence [5], and hereby postponing or even pre-empting the need for lifelong palliative ADT and its associated toxicity [9].

A recent systematic review showed that most published series on MDT are small with heterogeneous patient populations, making it difficult to make treatment recommendations [5, 7, 8]. To address these shortcomings, an international patient database on oligometastatic PCa recurrence (≤3 lesions) was established allowing to study a homogeneous patient population treated with stereotactic body radiotherapy (SBRT) [10]. A first analysis of this series showed that the majority of patients treated with SBRT for nodal recurrence, relapse within 2 years in nearby lymph node regions, with an estimated median time to progression of 12–18 months [11–13]. Similar results in terms of time to progression were observed in a large multi-institutional study exploring the role of salvage lymph node dissection (sLND) after nodal recurrence [14]. These comparable results could probably be explained by a lack of sufficient sensitivity of the current imaging techniques to perform a lesion-based approach, either by SBRT or sLND. An elective nodal irradiation approach using whole pelvic radiotherapy (WPRT) in addition to MDT could delay or even prevent such relapse [12, 15–17]. Improved progression-free survival (PFS) rates have been observed with WPRT in radiotherapy series [15–17], as well as after sLND as demonstrated in the retrospective study by Rischke et al. [12].

The current trial wants to explore the potential benefit in terms of metastasis-free survival (MFS) of an elective nodal approach by adding WPRT to either sLND or as alternative to focal SBRT. The proposed trial randomizes patients with oligorecurrent nodal PCa following primary PCa treatment to either MDT (sLND or SBRT) or WPRT plus MDT (focal radiotherapy boost or sLND). As two recent trials have suggested a progression-free and even overall survival benefit by adding temporary ADT to local therapy in case of biochemical recurrence [18, 19], a positive effect could also be expected for regional recurrence. Although the optimal duration of ADT is unknown, a minimal duration of 6 months of ADT seems advisable in this setting and is mandatory for both arms.

This trial will improve our insights in the pattern of recurrence following these treatment modalities with the expectation that WPRT will reduce the number of nodal relapses, improving MFS and postponing the need for palliative systemic treatment while maintaining quality of life (QoL).

## Methods & design

This study is approved by the Ethics committee of the Ghent University Hospital (EC/2018/0130) and is registered on both ClinicalTrials.gov (NCT03569241) and Swiss National Clinical Trials Portal (SNCTP000002947). This is a prospective multicentre randomized phase II trial designed for patients with PET-detected pelvic nodal oligorecurrence (≤5 nodes) following radical local prostate treatment (radical prostatectomy [RP], primary radiotherapy [RT] or both). In the standard arm A, patients will receive MDT by means of SBRT or sLND, combined with short-term ADT (6 months). In the experimental arm B, WPRT will be added to MDT (focal radiotherapy boost or sLND) and short-term ADT (6 months). It will be emphasized that the participation is voluntary and that the patient is allowed to refuse further participation in the protocol whenever he wants, which will not prejudice the patient’s subsequent care. Estimated study completion: December 31, 2023.

### Objectives

#### Primary endpoint

MFS: time between randomization and the appearance of a metastatic recurrence (any M1) as suggested by choline, FACBC or PSMA PET-CT/PET-MRI or death due to any cause. In case of biochemical progression, restaging will be performed, preferably with the same PET-tracer. In case of negative PET findings at biochemical relapse, repeated PET imaging should be performed on a 6-monthly basis or earlier if clinically indicated.

#### Secondary endpoints

- Clinical PFS: time between randomization and the appearance of a new recurrence (any N1 or M1) as suggested by PET-CT or PET-MRI, symptoms related to progressive PCa or death due to any cause.
- Local recurrence: evidence of a recurrence on imaging inside the surgical or radiotherapy field. Confirmation per biopsy is recommended. For radiotherapy schedules, local response and local progression is defined as per RECIST 1.1 criteria.
- Regional nodal recurrence: radiographic evidence (PET-CT or -MRI) of lymphadenopathy in the pelvis, outside the surgical or radiotherapy field. Histologic confirmation is recommended, especially in the absence of biochemical recurrence.
- Distant recurrence: appearance of distant metastases (M1a, M1b, M1c) outside the pelvis evidenced by PET-CT or -MRI.
- Biochemical PFS: time between randomization and the day of the first recorded biochemical progression (as defined below), clinical progression or death due to clinical progression.
- RP at diagnosis: biochemical recurrence = PSA > 0.20 ng/mL, confirmed 2 weeks later.
- Prostate radiotherapy at diagnosis: biochemical recurrence = PSA nadir + 2 ng/mL (Phoenix definition).
- Patients whose PSA does not drop below 0.20 ng/mL (if previous RP) or below the level before treatment (if previous prostate radiotherapy) at time of first response assessment at 3 months follow-up, are considered as non-responders to treatment and are considered to have a biochemical recurrence in case a second measurement at least 2 weeks later confirms a stable or rising PSA above this level.
- Time to start of hormonal treatment: time from trial randomization to start of hormonal treatment.
- Time to castration-resistant disease: time from trial randomization until castration resistant status as defined in the EAU-guidelines [1].
- PCa-specific survival: time from trial randomization until death due to PCa.
- Overall survival (OS): time from trial randomization until death from any cause.
- Acute and late toxicity due to radiotherapy will be assessed according to the National Cancer Institute Common Terminology Criteria for Adverse Events (CTCAE) version 4.0 [20], with special attention for rectal, gastro-intestinal and urinary adverse events. Surgery related morbidity, e.g. intra-operative complications (blood loss, injury to other pelvic organs …), and post-operative surgical complications will be scored using the Clavien-Dindo Classification of Surgical Complications [21].
- Acute toxicity: occurring during and up to 3 months after treatment completion.
- Late toxicity: occurring later than 3 months after treatment completion.
- Quality of life (QoL) scoring using the EORTC QLQ-C30 supplemented with QLQ-PR25. Raw scores will be transformed to a linear scale ranging from 0 to 100 according to the EORTC manual. The multi-item scales of the QLQ PR-25 will be analysed at both the individual item and the scale level. The results will be presented in accordance with recent guidelines for reporting health related QoL randomized controlled trials [22].
- Pattern of progression.
- Sensitivity/specificity of PET-imaging for the detection of nodal recurrences: limited to patients undergoing surgery.
- Biomarker discovery: to develop a miRNA panel predictive for treatment response using whole genome miRNA expression profiling [23].

### Inclusion criteria

- Histologically proven initial diagnosis of adenocarcinoma of the prostate.
- Biochemical relapse of PCa following radical local prostate treatment (RP, primary RT or RP +/− prostate bed [PB] adjuvant/salvage RT) according to the EAU guidelines 2019 [1].
- Following RP, patients with a biochemical relapse are eligible in case a nodal relapse is detected in the pelvis even in the absence of prior postoperative PB radiotherapy (adjuvant or salvage).
- In case of a suspected local recurrence following primary RT, a biopsy should confirm local recurrence. Patients with a confirmed local recurrence are eligible in case they also undergo a local salvage therapy.
- Nodal relapse in the pelvis on choline, PSMA or FACBC PET-CT or PET-MRI with a maximum of 5 positive nodal lymph nodes. The upper limit of the pelvis is defined as the aortic bifurcation.
- WHO performance state 0–1
- Age ≥ 18 years
- Absence of any psychological, familial, sociological or geographical condition potentially hampering compliance with the study protocol and follow-up schedule; those conditions should be discussed with the patient before registration in the trial.
- Before patient registration/randomization, written informed consent must be given according to ICH/GCP and national/local regulations.

### Exclusion criteria

- Bone or visceral metastases
- Para-aortic lymph node metastases (above the aortic bifurcation)
- Local relapse in the prostate gland or bed not suitable for curative treatment
- Previous irradiation of the pelvic and/or para-aortic nodes
- Serum testosterone level < 50 ng/dL or 1.7 nmol/L at time of randomization
- Symptomatic metastases
- Lymph node metastases in previously irradiated areas resulting in dose constraint violation
- Contraindications to pelvic radiotherapy (e.g. chronic pelvic inflammatory bowel disease)
- Contraindications to ADT
- PSA rise while on active treatment with ADT (LHRH-agonist, LHRH-antagonist, anti-androgen, estrogen)
- Previous treatment with a cytotoxic agent for PCa
- Treatment during the past month with products known to influence PSA levels (e.g. fluconazole, finasteride, corticosteroids …)
- Other active malignancy, except non-melanoma skin cancer or other malignancies with a documented disease-free survival for a minimum of 3 years before randomization.
- Patients can only be randomized in this trial once.

### Pre-randomization and evaluation

Patients must be restaged within 8 weeks prior to randomization with either a whole-body choline, PSMA or FACBC PET-CT/−MRI to exclude M1 disease (para-aortic node(s), bone or visceral metastases). Additional imaging modalities to confirm or to rule out M1 disease are optional. For patients without prior RP it is recommended to perform a multiparametric MRI of the prostate to rule out local recurrence. Local relapses must be biopsy proven. The following pre-treatment work-up, which is also depicted in Table 1, must be performed:

**Table 1:** Summary of study visits and procedures

TIMEPOINT				STUDY PERIOD						
	Screening		Allocation	Treatment period			Follow-up period			
	Within 8 weeks prior to randomization	Within 2 weeks prior to randomization	Baseline	Start	During	End	1 month after treatment ± 7 days	3 months after treatment ± 14 days	6 months after treatment ± 14 days	6-monthly, yearly, until progression (g) ± 14 days
Window										
**ENROLMENT:**										
**Eligibility screen**	x									
**Signed ICF**	x									
**Randomization**			x							
**INTERVENTIONS (a,b):**										
**If sLND**				x	x	x				
**If WPRT**				x	x	x				
**If SBRT**				x	x	x				
**ADT**				x	x	x	x	x	x	
**ASSESSMENTS:**										
**Medical history**	x									
**Patients characteristics**		x								
**Physical exam (c)**		x				x	x	x	x	x
**Lab**										
PSA		x				x (h)	x	x	x	x
Testosterone		x					x	x	x	x
Biomarker		x								x (i)
**Imaging**										
PET-CT (d)	x							(x)	(x)	(x)
MRI (e)	x									
**Adverse Events**										
Baseline toxicity		x								
Acute toxicity (f)				(x)	(x)	x	x	x		
Late toxicity									x	x
**Medication**										
Prior medication		x								
Con med’s						x	x	x	x	x
**Quality of life**										
QLQ-C30		x						x	x	x
QLQ-PR25		x						x	x	x
**Survival status**							x	x	x	x

(a) All arms should receive an LHRH-agonist or antagonist for a duration of 6 months using 1 monthly formulations. In case of SBRT/WPRT, ADT should start no later than the first fraction (whichever RT treatment comes first) and no earlier than 2 weeks before the start of radiotherapy. In case of sLND, ADT should be started no earlier than 1 day postoperatively and no later than 10 days postoperatively

(b) Treatment should start preferably within 4 weeks after randomization, but no later than 8 weeks after randomization. Treatment period is defined as the time between first treatment day and last treatment of surgery and/or radiotherapy

(c) Physical examination including scoring of Performance Status. Weight and height will only be measured at screening

(d) Whole body choline, PSMA or FACBC PET-CT/−MRI. During follow-up repeat PET-CT only at time of biochemical relapse and then 6-monthly afterwards until clinical progression is determined or earlier in case of symptomatic progression

(e) Optional: for patients without a prior radical prostatectomy it is recommended to perform a multiparametric MRI of the prostate to rule out local recurrence

(f) During the treatment period a toxicity assessment should be done at least once a week in case of WPRT. For SBRT toxicity assessment should be done only at end of treatment, for sLND at discharge (Clavien-Dindo scale)

(g) 6-monthly follow-up for a minimum of 24 months, with yearly follow-up thereafter up to 60 months or until progression. In case of clinical progression, the patient will be treated according to the EAU guidelines

(h) In case of SBRT/WPRT, PSA has to be determined on the day of the last fraction. In case of sLND in arm A, PSA will be determined at 4 weeks after surgery (follow-up month 1). In case of sLND + WPRT in arm B, PSA will be determined at 4 weeks post-WPRT (follow-up month 1)

(i) At time of primary endpoint is reached, a new biomarker sampling is preferred, but optional

#### Within 8 weeks prior to randomization

- Signed ICF
- Whole-body choline, PSMA or FACBC PET-CT/PET-MRI
- Medical history (including previous therapies and PSA values)
- In- and exclusion criteria

#### Within 2 weeks prior to randomization

- Patient characteristics
- Baseline symptoms (including gastrointestinal and genitourinary morbidity) and medication
- QoL questionnaires (QLQ-C30, QLQ-PR25)
- Physical examination including scoring of Performance Status, weight and height
- Baseline pre-treatment PSA and testosterone determination
- Biomarker collection

### Randomization

**Fig. 1:**
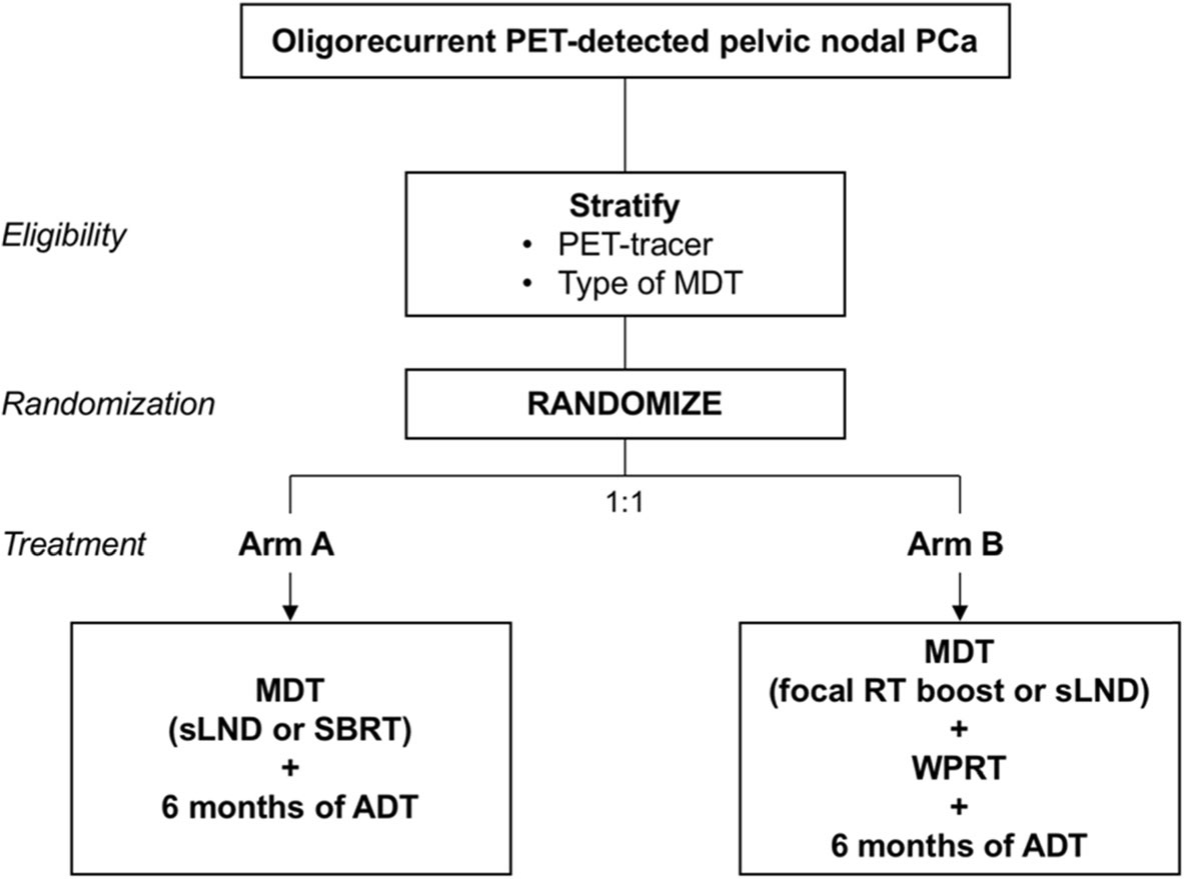
Trial design. **PCa** = prostate cancer, **MDT** = Metastasis-directed therapy, **sLND** = salvage lymph node dissection, **SBRT** = stereotactic body radiotherapy, **ADT** = androgen deprivation therapy, **WPRT** = whole pelvic radiotherapy, **RT** = radiotherapy

Treatment will be allocated at random in a 1:1 ratio using Clinsight® online randomization system. Patients will be stratified according to the type of PET-tracer used (choline, PSMA or FACBC) and the type of MDT (SBRT or sLND) (Fig. 1). As this is an open label trial, randomization will not be blinded.

Patients will be randomized to receive one of the following treatments (Fig. 1):

**Arm A:**

- sLND + 6 months of ADT or
- SBRT (30 Gy in 3 fractions) + 6 months of ADT

**Arm B:**

- sLND + WPRT (45 Gy in 25 fractions) + 6 months of ADT or
- WPRT with simultaneous integrated boost (45 Gy in 25 fractions with SIB up to 65 Gy) + 6 months of ADT

### Evaluation during treatment

***Following sLND:***

- Physical examination including Performance score at discharge
- PSA measurement at the first postoperative visit (4 weeks)
- Toxicity assessment using Clavien-Dindo scale at discharge
- Concomitant medication
- Operating characteristics: surgical technique, operating time, blood loss, number of nodes removed per lymph node region, number of positive nodes per lymph node region, indicate lymph node regions sampled.
- Nodes should be sent in separate containers for histopathological analysis.

***WPRT or SBRT:***

- Physical examination at the end of treatment
- Acute toxicity assessment (once a week for WPRT; for SBRT at the end of treatment): the highest grade of adverse events during radiotherapy will be reported at the end of treatment.
- Concomitant medication
- Treatment planning
- Record planning results

### Follow-up

Follow-up is planned at month 1, 3 and 6 following treatment completion, 6-monthly thereafter until month 24 and then yearly up to month 60. Patients who withdraw from trial treatment in the absence of progression should continue to have follow-up visits as defined above. The following investigations should be performed at every follow-up visit (Table 1):

- Clinical examination with late toxicity scoring
- PSA and testosterone measurement
- QoL-scoring
- PET-imaging only at time of biochemical progression or symptomatic progression and then 6-monthly thereafter until clinical progression is determined. In case of biochemical progression, the PSA should at least be 0.50 ng/mL before a new PET-CT scan should be considered. The location of progression should be recorded.
- At time of primary endpoint is reached, a new biomarker sampling is preferred, but optional.
- Concomitant medication
- Survival status

In case of clinical disease progression the patient will be treated according to each centre policy, but adherence to the EAU guidelines [24] is recommended. In case of local or regional oligometastatic recurrence during follow-up, a new MDT is allowed (this treatment falls outside the scope of this protocol).

### Interventions

#### General information

All patients will be presented at the multidisciplinary urology tumour board prior to treatment. The choice of MDT (SBRT or sLND) will depend on the localization and size of the metastases, the nearby organs at risk (OAR) and previous treatment in the vicinity of the metastases.

In case of a biopsy-confirmed local recurrence a local therapy should be provided for a patient to be eligible. Local radiotherapy to the PB is highly recommended in case of pT3a/b-4 and/or R1 disease, or in case of a PSA level ≥ 2 ng/mL, even if imaging rules out a local relapse. In case of randomization to arm A, PB-RT can either follow sLND or SBRT, or can be combined with SBRT. In case of randomization to arm B, PB-RT radiotherapy should be combined with WPRT.

#### Radiotherapy

1. Positioning and CT simulation:
  CT simulation has to be performed in supine position by using an individualized immobilization device in the treatment position on a flat table. The use of i.v. contrast is highly recommended, as well as the use of leg and knee support. It is recommended that patients are scanned and treated with comfortably full bladder and for PB-RT with an empty rectum. If an MRI is used for co-registration (recommended for PB-RT), the same position for both the planning CT and MRI is recommended. The position of the patient will be reproduced using skin marks and orthogonal laser beams during treatment preparation and execution.

- For WPRT or PB-RT: CT slice thickness should be 5 mm or less. The planning CT should include at least the pelvis from the lower part of the second lumbar vertebra (L2) to the lower part of the ischial tuberosities. The entire target volume and all OAR must be included in the CT scan. The clinical target volume (CTV), planning target volume (PTV) and OAR must be delineated on all CT slices in which these structures are visible.
- For SBRT: CT slice thickness should be 3 mm or less. The planning CT scan should extend at least 10 cm superior and inferior beyond the treatment field borders. Multiple isocentres and a sum plan might be necessary for multiple suspicious nodes. A CBCT prior to every treatment-isocentre is needed.

2. Organs at risk:
  Delineation of OAR should be according to the RTOG guidelines. Dose constraints to OAR for WPRT are in agreement with the QUANTEC guidelines [25–27] and the doses to OAR for SBRT are stipulated in the AAPM report 101–3 fractions schedule [28].
3. Definition of target volumes:

- The gross target volume (GTV) will be defined as all known gross disease before any treatment, defined by CT/MRI/PET-CT images and/or clinical information. A GTV_PB may be defined with the help of MRI and PET imaging.
- The CTV will encompass regions at risk of microscopic extension. For CTV_PB, consensus guidelines such as EORTC, RTOG or FROGG are being followed [29–31]. The CTV_LNN consists of the pelvic lymph node regions as described in the RTOG guidelines [31], with the exception that delineation of the common iliac lymph nodes should start at the L4/L5 interspace [32] and should encompass the GTVs. No CTV is mandatory for SBRT.
- The PTV (PTV_PB, PTV_SBRT and PTV_LNN) will provide margin around the CTV to compensate for variability in daily treatment set-up and internal CTV motion due to breathing or motion during treatment. PTV_PB is created by a 5 to 10 mm margin for centres with or without image-guided radiotherapy, respectively. The PTV_LNN must include the entire CTV_LNN plus a minimum 3D-margin of 5 mm. For the nodal GTVs located within CTV_LNN, a margin of 5 mm is used. The PTV_SBRT should encompass the GTV with a minimal margin of 3 mm in all directions.

4. Dose prescription:
  Intensity modulated radiotherapy (IMRT) or use of rotational techniques is mandatory in case of WPRT and SBRT. In case of SBRT, treatment with Cyberknife® is allowed. 3D conformal technique is not allowed in this trial.

- WPRT or PB-RT:
  All fields and the entire PTV_LNN must be treated daily for 5 days per week in a once-daily schedule of 1.8 Gy per fraction, for a total dose of 45 Gy in 25 fractions. The GTV should receive an integrated boost to a median dose of 65 Gy in 25 fractions, 2.6 Gy per fraction. If a suspicious node is still present following sLND, it is allowed to boost the GTV to the same dose during WPRT. In case of PB-RT, the PTV_PB should receive at least 66 Gy in 33 fractions.

Normalization of the treatment plan will cover 95% of the PTV_LNN or PTV_PB with the prescription dose. Furthermore, 98% of the PTV_LNN or PTV_PB should receive 95% of the prescription dose with 2% of the PTV not exceeding 107% of the prescribed dose.

- SBRT (MDT):
  A total dose of 30 Gy (80% of the maximal dose) will be delivered in 3 fractions and fractions will be separated > 48 h and < 96 h. Treatment will be prescribed to the periphery of the target (80% of the dose (= 30 Gy), should cover 90% of the PTV). In case of violation of dose constraints to the surrounding OAR, the prescription will be adapted accordingly.

#### Surgery – sLND (MDT)

A bilateral extended lymph node dissection of the true pelvis will be performed either via an open or minimally invasive approach, which is at the discretion and expertise of the surgeon. A minimally invasive technique is preferred, but not mandatory. In case of pelvic nodal uptake, all nodal/fibrofatty tissue at the external and internal iliac regions and obturator fossa region will be removed. For pelvic nodal metastases, in case of a previous extended lymphadenectomy, only the suspicious lymph node(s) will be removed. The definition of the true pelvis is defined according to the AJCC Cancer Staging Manual 7th edition and the regions are defined by Mattei et al. [33]. An extended lymph node dissection of regions I and II is recommended in this study.

In case of WPRT after sLND in arm B: WPRT should start between the 4th and 12th week following sLND. In case the morbidity of sLND does not allow a safe start of RT in this period, radiotherapy will not be carried out.

#### Androgen deprivation therapy

All arms should receive an LHRH-agonist or -antagonist for a duration of 6 months using 1-monthly formulations. In case of LHRH-agonist, flare prevention with an anti-androgen is recommended for at least 5 days prior to the first injection of the agonist and should not be continued for longer than 15 days of the first month duration. ADT-related toxicity should be managed according to Nguyen et al. [34].

- In case of sLND, ADT should be started between day 1 and day 10 postoperatively.
- In case of SBRT/WPRT, ADT should start no later than the first day of WPRT and no earlier than 1 week before the start of SBRT/WPRT.

Palliative ADT should not be started for biochemical progression without documented clinical progression. In case of symptomatic progression, palliative ADT is mandatory. In case of clinical asymptomatic progression, delayed ADT until progression to a symptomatic state is allowed in well informed men (EAU 2019 guidelines). In general, starting ADT in asymptomatic patients is recommended only if conventional imaging confirms clinical progression. The start of ADT for PET-positive lesions not suspicious on conventional imaging (CT/MRI/bone scintigraphy) is not recommended. In case of clinical oligometastatic progression, repetition of MDT is allowed for all arms.

### Statistical analysis

#### Sample size

This prospective randomized phase II design aims to determine whether the treatment arm can be tested in a subsequent phase III trial. Patients will be randomized (1:1) to receive either arm A: MDT (sLND or SBRT) plus ADT or arm B: WPRT plus MDT (focal radiotherapy boost or sLND) plus ADT. Stratification is according to the type of MDT and the type of PET-tracer. The sample size is based on the stratified Log-rank test. A scenario analysis has been performed to reflect the different and reasonable possible values of the parameters. The two-sided significance level alpha was set at 0.20 and the power maintained at 80%. In this chosen scenario, the median MFS following MDT has been estimated as 24 months in arm A. Assuming uniform accrual over 48 months with 24 months additional follow-up time, a total of 178 patients are needed to detect a 12-month difference in median MFS from 24 to 36 months, considering a 5% rate of loss to follow-up. The total study length will be 72 months.

#### Data analysis

Patients will be analysed in the group to which they are assigned. The probability of MFS, PFS and OS will be estimated using the Kaplan-Meier Survival Analysis method. Stratified log-rank tests will be performed to compare PFS and OS between the two treatment arms. Cox proportional hazards models will be fit to assess the effects of treatment and baseline clinical and pathologic features (such as PSA, PSA doubling time, Gleason score etc.) on MFS, PFS or OS. All *p*-values are set at 0.20 for primary analysis and at 0.05 for other analyses. Statistical analysis will be performed with SPSS version 25 (IBM, Armonk, NY, USA) and R version 3.5.3.

### Data management and monitoring

Study data will be stored in a digital archive developed in cooperation with Methodology, Biostatistics and Data Management unit, Centre Georges François Leclerc, Dijon, France, that will make use of a clinical trial compatible eCRF based on the web-based data capture system Clinsight®. Current international requirements for data protection will be followed. Health related personal data captured during this project are strictly confidential and accessible only by investigators and authorized personnel; disclosure to third parties is prohibited. Coding will safeguard participants’ confidentiality. No on-site quality control or audits will be foreseen.

In order to avoid introducing bias, no interim analysis will be performed. There is no anticipated harm and compensation for trial participation. Serious adverse events will be reported to the UZ Ghent Ethical Committee and the local Ethics Committee and must be followed up for outcome. After completing the trial, participants will be followed by the radiation oncologist and urologist.

### Translation research

In patients willing to provide a blood sample, we aim to collect both serum and plasma samples at baseline in a standardized manner. These samples will be used to identify potential prognostic or predictive biomarkers that might help us identify the ideal patient for different approaches. In patients undergoing sLND, FFPE tissue can be compared with the blood-based markers. Depending on the evolution of the different techniques over the trial period, specific biomarkers will be evaluated among others, but not limited to miRNA, cfDNA, cfRNA.

### Radiotherapy quality assurance

Quality assurance (QA) for this multicentre trial follows the nomenclature of The Global Clinical Trials Quality Assurance of Radiation Therapy Harmonization Group (GHG) [35] and the Radiation Therapy Quality Assurance (RTQA) procedures of the European Organisation for Research and Treatment of Cancer (EORTC) [36]. The QA consists of a site and study specific questionnaire (SSQ) encompassing a facility questionnaire and a proof of beam output audit. A two-phase benchmark case is run. In phase I participating centres submit the delineation of a SBRT and WPRT-case for comparison to reference contours. In phase II the centres perform a dose-planning of a SBRT and WPRT-case using reference contours and verify the delivery with a complex dosimetry check according to the institution’s clinical practice. No individual case review is foreseen in this trial.

### Participating sites

This multicentre study is currently conducted at following sites:

- Belgium: Ghent University Hospital; Iridium Cancer Network, GZ Antwerp; AZ Groeninge, Kortrijk; Leuven University Hospital; Jules Bordet Institut, Bruxelles; AZ Maria-Middelares, Ghent.
- Switzerland: Geneva University Hospital; University Hospital Basel; Bern University Hospital; University Hospital Zürich; Kantonsspital St. Gallen.
- Spain: Cruces University Hospital, Barakaldo; University Hospital La Princesa, Madrid; Hospital Universitari i Politècnic la Fe, Valencia.
- Italy: Humanitas Research Hospital Milan.
- Norway: Oslo University Hospital.
- Australia: Epworth Healthcare, Melbourne.

All participating centers have experience in research within the urological oncological field. Data collection in these hospitals enables the study to include enough patients and gives the study sufficient power.

## Discussion

The entity regional nodal oligorecurrent PCa is not mentioned in the treatment guidelines but is an emerging clinical situation since the introduction of new molecular imaging techniques, such as choline and more recently PSMA PET-CT, in the restaging of recurrent PCa [2–4]. As there are no specific treatment recommendations for patients with limited metastatic disease, different treatment approaches are currently used, mostly by means of MDT (sLND or SBRT) to delay disease progression and to postpone androgen ablation treatments [5, 6]. Although MDT approaches represent potential effective treatment options for oligorecurrent patients according to the APCCC expert consensus meeting [37, 38], available treatment results are mostly based on retrospective series, including an overall low number of treated patients with a limited follow-up and several heterogeneities in the treatment modalities proposed. The best treatment approach for patients with oligorecurrent nodal disease remains therefore debated.

The only prospective data available in literature have been reported by a single centre non-randomized study (POPSTAR) [39] and a randomized phase II trial (STOMP) [40], both using SBRT. SBRT directed to the single nodal relapse has the advantage to deliver highly conformal irradiation with very high radiotherapy doses in only few fractions (3 to 10) over a short overall treatment time (OTT). Major potential benefits of this technique arise from excellent local tumour control, a negligible acute toxicity, an excellent patient’s compliance, the possibility for repeated SBRT treatments for outfield relapses and a possible immune-mediated impact on the tumour growth outside the RT field (abscopal effect). For sLND, only retrospective data are available. The expected benefit of this technique is the pathological validation of removed lymph nodes and the removal of false PET-negative lymph nodes in a predefined elective area of the pelvis. Fossati et al. observed in a recently published series that in patients with ≥3 positive spots on choline or PSMA PET/CT at time of recurrence, the number pathologically involved nodes detected by sLND was twice as high [14]. Salvage pelvic LND however is an invasive procedure with potential complications and long-term morbidity, even in experienced centres.

Although sLND is not a pure lesion-directed approach, the recurrence patterns of both SBRT and sLND are comparable with > 60% of patients relapsing in adjacent lymph node regions [11–13]. This might be explained by the fact that even the most extended sLND does not cover all potential pelvic lymph node metastasis landing sites. In a previous paper from our group, we indicated that an extended sLND technique only covers around 50% of these sites [41]. Consequently, we hypothesize that a larger RT field covering the elective nodal regions in a treatment field may be a more effective alternative to focal strategies. This is supported by a retrospective study by Rischke et al. [12], indicating a reduced pelvic lymph node recurrence rate following sLND plus WPRT as compared to sLND alone. On the other hand, compared to MDT strategies, longer OTT, higher toxicity rates and limited re-treatment possibilities are expected by delivering elective nodal irradiation. Nevertheless, WPRT grade 3 toxicity rates appear to be low in recent trials such as OLIGOPELVIS and SPPORT [42, 43].

In both arms, short term ADT will be mandatory. The rationale for the use of temporary ADT is extrapolated from the data in the salvage setting (GETUG16, RTOG9601, SPPORT, OLIGOPELVIS), indicating that temporary ADT reduces the rate of biochemical and clinical recurrences [12, 18, 19, 42, 43]. There are no clear guidelines on the ideal duration of ADT in this setting, so we opted for 6 months as studied in GETUG16, OLIGOPELVIS and SPPORT [19, 42, 43].

As no direct comparison of the two approaches have been made to date, prospective studies using well-defined endpoints and predefined treatment approaches are therefore needed to validate the best treatment approach in the nodal oligorecurrent setting. The STORM study will be the first prospective multicentre randomized controlled phase II trial to assess the possibility of prolonging the MFS by adding WPRT to either sLND or SBRT, both in combination with short-term ADT in PCa patients with nodal oligorecurrent disease.

## Supplementary information

**Additional file 1.**

## Data Availability

The datasets generated during the current study are not publicly available due since they will contain patient data and the Informed Consent does not include sharing data publicly. The datasets are available from the corresponding author on reasonable request. All data generated or analyzed during this study will be included in the published article.

## Abbreviations

AAPM: American Association of Physicists in Medicine
ADT: Androgen Deprivation Therapy
CBCT: Cone beam computed tomography
cfDNA: Circulating cell-free DNA
cfRNA: Circulating free RNA
CRPC: Castrate Resistant Prostate Cancer
CT: Computed tomography
CTCAE: Common Terminology Criteria for Adverse Events
CTV: Clinical Target Volume
DNA: Deoxyribonucleic acid
EAU: European Association of Urology
EORTC: European Organization for Research and Treatment of Cancer
FACBC: Anti-1-amino-3-18F-fluorocyclobutane-1-carboxylic acid
FFPE: Formalin-Fixed Paraffin-Embedded
FROGG: Faculty of Radiation Oncology Genito-Urinary Group
GCP: Good Clinical Practice
GTV: Gross Tumour Volume
Gy: Gray
ICF: Informed Consent Form
ICH: International Conference on Harmonization
IMRT: Intensity modulated radiotherapy
LHRH: Luteinizing Hormone-releasing Hormone
LNN: Lymph Nodes
MDT: Metastasis-Directed Therapy
MFS: Metastasis-free survival
miRNA: microRNA
MRI: Magnetic Resonance Imaging
NCI: National Cancer Institute
OAR: Organs at Risk
OS: Overall Survival
PB: Prostate Bed
PCa: Prostate Cancer
PET-CT: Positron Emission Tomography-Computed Tomography
PFS: Progression-free Survival
PSA: Prostate Specific Antigen
PSMA: Prostate Specific Membrane Antigen
PTV: Planning Target Volumes
QLQ: Quality of Life Questionnaire
QoL: Quality of life
QUANTEC: Quantitative Analyses of Normal Tissue Effects in the Clinic
RECIST: Response Evaluation Criteria In Solid Tumors
RNA: Ribonucleic acid
RP: Radical prostatectomy
RT: Radiotherapy
RTOG: Radiation Therapy Oncology Group
SBRT: Stereotactic Body Radiotherapy
SNCTP: Swiss National Clinical Trials Portal
sLND: salvage Lymph Node Dissection
WHO: World Health Organization
WPRT: Whole pelvic radiotherapy
