# Supplementary material for: PEACE V – Salvage Treatment of OligoRecurrent nodal prostate cancer Metastases (STORM): a study protocol for a randomized controlled phase II trial
Source: BMC Cancer. 2020 May 12;20:406. doi: 10.1186/s12885-020-06911-4 (PMC7216526; doi:10.1186/s12885-020-06911-4)
Supplement: Supplementary file 1 — Additional file 1. [file 12885_2020_6911_MOESM1_ESM.docx]

**Institutional Review Boards (IRB)**

| **Institutional Review Board (IRB) - name** | **Local PI** | **Address** |
| --- | --- | --- |
| Commissie voor Medische Ethiek, Bimetra Clinics  *(Central IRB)* | Prof. Dr. P. Ost | Ghent University Hospital, Ghent, Belgium; lead by Prof. dr. D. Matthys |
| Ethisch Comité | Dr. F. Ameye | AZ Maria-Middelares, Kortrijksesteenweg 1026, 9000 Ghent, Belgium |
| Commissie Medische Ethiek GZA | Prof. Dr. P. Dirix | Iridium Cancer Network, GZA, Oosterveldlaan 24, 2610 Wilrijk, Belgium |
| Ethische Commissie Onderzoek UZ/KU Leuven | Prof. Dr. W. Everaerts | University Hospital Leuven, Herestraat 49, 3000 Leuven, Belgium |
| Ethisch comité | Dr. N. Liefhooghe | AZ Groeninge Kortrijk, President Kennedylaan 4, 8500 Kortrijk, Belgium |
| Comité d’Ethique | Dr. F. Otte | Jules Bordet Institute/AMPR, Waterloolaan 121, 1000 Brussel, Belgium |
| Regional Ethics Committee | Prof. Dr. T. Zilli | Geneva University Hospital, Geneva, Switzerland |
| Clinical Ethics | Dr. A. Papachristofilou | University Hospital Basel, Basel, Switzerland |
| The Cantonal Research Ethics Commission | Dr. M. Shelan | Bern University Hospital, Bern, Switzerland |
| Clinical ethics | Prof. Dr. M. Guckenberger | University Hospital Zürich, Zürich, Switzerland |
| Ethics Committee | Dr. P.M. Putora | Kantonsspital St. Gallen, St. Gallen, Switzerland |
| Clinical Research Ethics Committee | Dr. A. Gomez-Iturriaga | Cruces University Hospital, Barakaldo, Spain |
| Ethics Committee | Dr. A. Zapatero | University Hospital La Princesa, Madrid, Spain |
| Ethics Committee | Dr. F. Couñago | University Hospital of Quirón, Madrid, Spain |
| Research Ethics Committee | Dr. A. Conde-Moreno | Hospital Universitari i Politècnic la Fe, Valencia, Spain |
| Ethics Committee | Dr. M. Scorsetti | Humanitas Research Hospital Milan, Italy |
| Centre for Medical Ethics | Dr. R. Heikkilä | Oslo University Hospital, Oslo, Norway |
| Melbourne Health Human Research Ethics Committee | Prof. Dr. S. Siva | Epworth Healthcare, Melbourne, Australia |
